# Meta‐Analysis of the Use of Chinese Martial Arts Training for Alleviating Cancer‐Related Fatigue in Cancer Survivors

**DOI:** 10.1002/cam4.71676

**Published:** 2026-03-10

**Authors:** Benjamin K. K. Lau, Tai Wa Liu, Shamay S. M. Ng, William W. N. Tsang

**Affiliations:** ^1^ Department of Rehabilitation Sciences The Hong Kong Polytechnic University Hong Kong SAR China; ^2^ School of Nursing and Health Sciences Hong Kong Metropolitan University Hong Kong SAR China; ^3^ Research Centre for Chinese Medicine Innovation The Hong Kong Polytechnic University Hong Kong SAR China

## Abstract

Cancer was the second leading cause of death worldwide in 2018 according to WHO. The disease burden continues to grow and has tremendous impacts on families and healthcare systems. Cancer‐related fatigue is one of the most distressing symptoms experienced by cancer patients and has adverse impacts on the patients' quality of life and functioning. Both pharmacological and non‐pharmacological interventions could be adopted to tackle cancer‐related fatigue. Among non‐pharmacological interventions, exercise training is recommended by various authorities, such as the American College of Sports Medicine and the National Comprehensive Cancer Network, to manage cancer‐related fatigue. In particular, resistance training with moderate‐intensity exercise has been proven to be the most effective intervention for alleviating cancer‐related fatigue. Chinese martial art that includes moderate‐intensity physical training with a strong mind–body component is believed to offer mental well‐being and stress reduction benefits in addition to the benefits of traditional resistance training, thus potentially enhancing the overall quality of life of cancer patients. This systematic review and meta‐analysis aimed to identify the effectiveness of Chinese martial arts training in reducing cancer‐related fatigue in cancer patients. Sixteen randomised controlled trials (RCTs) with 1365 cancer patients were included in this systematic review and meta‐analysis. All of the included studies had implemented either Tai Chi or Baduanjin as the martial arts training intervention. The results of the meta‐analysis showed that the overall effects of the trainings were not significant (standardised mean difference [SMD]: −0.23, 95% confidence interval [CI]: −0.57 to 0.11, *p* = 0.19). In the sub‐group analysis, martial arts training administered over a shorter intervention period (less than 12 weeks) was found to yield a significant medium‐to‐large pooled effect size on the reduction of cancer‐related fatigue (SMD: −0.77, 95% CI: −1.54 to −0.01, *p* = 0.05).

**PROSPERO Registration Number:** CRD42023416590

## 1 Introduction

Cancer‐related fatigue is one of the most distressing symptoms that cancer survivors experience during the course of their disease. It typically presents as persistent and intense exhaustion that is out of proportion and can greatly impair a patient's capacity to carry out daily tasks, sustain social connections, and participate in work or hobbies [1]. This condition impacts physical well‐being and also carries substantial psychological and social consequences, often resulting in heightened depression, anxiety, and social withdrawal. It has serious impacts on the quality of life of cancer survivors and leads to a loss of productivity for both the patients and their caregivers. The socioeconomic burden of this condition is substantial.

As pharmacological intervention is concerned, the classes of medications used to alleviate cancer‐related fatigue include steroids, such as dexamethasone, psychostimulants, for example methylphenidate and modafinil, and anti‐depressants, for instance Bupropion. In a recent clinical trial, a new drug called ponsegromab was found to be effective in reducing cancer cachexia among patients with elevated levels of growth differentiation factor 15 (GDF‐15), which is a circulating cytokine [2]. As cachexia is one of the contributing factors for cancer‐related fatigue, patients receiving ponsegromab in this study were found to have reduced fatigue levels. While the registration‐enabling studies are still underway, this drug may have huge impacts in the future interventions of cancer‐related fatigue.

As non‐pharmacological interventions are concerned, exercise training has proven to be effective in reducing cancer‐related fatigue. The effects of martial arts training, as a form of exercise, have also been investigated in recent years. Martial arts originated in the Orient and have been practised for hundreds of years. They include offensive and defensive combat systems and have commonly been modified for sports, self‐defence and recreation in modern decades [3]. Of the different schools of martial arts, karate and taekwondo place emphasis on striking with fists and feet. Judo and jujitsu are Japanese styles that utilise joint locking, grappling and throwing techniques [3]. The fighting and combat form of martial arts is not the main focus of this meta‐analysis. Instead, this study focuses on the use of Chinese martial arts that incorporate mind–body components, such as Tai Chi and Qigong, for decreasing cancer‐related fatigue.

Chinese martial arts with mind–body components involve a range of gentle and specific movements, together with the purposeful regulation of breath and mind and body coordination [4]. The practice of Tai Chi is beneficial not only for enhancing cardiorespiratory health and balance control but also for reducing anxiety and improving mood and self‐esteem in healthy older adults [5]. Tai Chi can be regarded as a complementary approach to reduce fatigue and improve well‐being in cancer patients [6]. In recent decades, several clinical studies have implemented mind–body exercises to treat cancer‐related fatigue. Tai Chi also incorporates a resistance training component in which one utilizes his or her body weight as resistance [5]. Research evidence suggests that moderate‐intensity resistance training is effective in reducing cancer‐related fatigue. Thus, this study focused on the potential benefits of practicing Chinese martial arts with a resistance training component for alleviating cancer‐related fatigue in prostate cancer patients.

A systematic review and meta‐analysis conducted by Wayne et al. [7] revealed that Tai Chi and Qigong training significantly alleviated cancer‐related fatigue. Of the studies included in their meta‐analysis, 12 studies measured fatigue, 10 of which were randomised controlled trials (RCTs). Only the data from these RCTs were pooled for analysis. The overall effect size was found to be −0.53 (Hedges' *g* = −0.53, 95% confidence interval [CI]: −0.97 to −0.28, random‐effects model, *p* < 0.001). In the subgroup analysis, Tai Chi and Qigong were found to be beneficial compared with active controls, which included sham Qigong, low‐impact exercise and a group line‐dancing programme (Hedges' *g* = −0.48, 95% CI: −0.98 to −0.14, *p* = 0.009), and a no‐treatment control (Hedges' *g* = −0.75, 95% CI: −1.35 to −0.14, *p* = 0.016).

A systematic review and meta‐analysis conducted by Sur et al. [8], which included 17 RCTs with 1103 patients, evaluated the effects of martial arts on cancer‐related fatigue and quality of life in cancer patients. The control groups in the included studies included active control, e.g., cognitive behavioural therapy, line‐dancing, sham Qigong and psychological support, and inactive control, namely waitlist control and usual care. The effects of martial arts, in terms of the outcome measures used, were analysed in different subgroups. The results showed that martial arts significantly reduced cancer‐related fatigue, as measured using the Functional Assessment of Chronic Illness Therapy—Fatigue (FACIT‐F) (standardised mean difference [SMD] = 0.68, 95% CI: 0.39–0.96; *p* < 0.001) and Multidimensional Fatigue Symptom Inventory Short Form (MFSI‐SF) (SMD = −0.51, 95% CI: −0.80, −0.22; *p* = 0.0005).

In contrast, a meta‐analysis conducted by Yin et al. [9] that aimed to evaluate the effectiveness of Qigong practice on self‐reported cancer‐related fatigue among cancer patients showed inconclusive results. The study included 13 RCTs with 1154 cancer patients. Specifically, the results showed that Qigong had a significant effect in terms of reducing cancer‐related fatigue compared with usual care or waitlist control (Hedges' *d* = 0.66, 95% CI: 0.07–1.26, *p* < 0.001). However, in the subgroup analysis, the effect of Qigong was not significant when compared with Western exercise (Hedges' *d* = 0.46, 95% CI: −0.02 to 0.95, *p* = 0.06) or no‐treatment control (Hedges' *d* = 0.10, 95% CI: −0.23 to 0.43, *p* = 0.60).

## 2 Objective of the Literature Review

The abovementioned reviews and meta‐analyses have not evaluated outcomes from a clinical perspective, e.g., the timing of implementation of martial arts training and effects of various treatment durations. This meta‐analysis aimed to provide up‐to‐date qualitative and quantitative analyses of the effects of martial arts training on cancer‐related fatigue from a clinical perspective. The results are expected to provide clinical guidelines for future clinical practice.

## 3 Methodology

### 3.1 Search Strategy

Seven electronic databases, namely CINAHL, CNKI, Cochrane Library, Embase, PsycINFO, PubMed, and Web of Science, were searched for relevant studies from 1 March to 30 April 2023. The keywords used for the search were Qi Gong/Qigong/Ba duan jin/Baduanjin/Eastern exercise/Tai Chi/Taichi/Tai Chi Chuan/Tai‐ji/Taiji/Taijiquan/Wing Chun/Wing Tsun/Ving Tsun/Ving Chun/Martial art AND Cancer/Neoplasm/Tumour/Tumour AND Cancer‐related fatigue/level of fatigue/fatigue.

**FIGURE 1:**
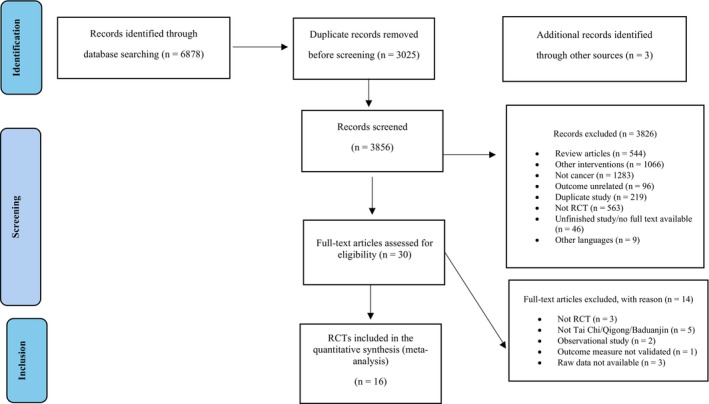
PRISMA flow diagram.

Additional relevant studies were also retrieved from the bibliographies of the extracted articles, systematic reviews and meta‐analyses by manual searching. The searches yielded studies published from 2010 to 2022. The PRISMA flow diagram of study selection is provided in Figure 1.

This systematic review and meta‐analysis has been registered in PROSPERO, an international prospective register of systematic reviews (ID: CRD42023416590) (Table 1).

**TABLE 1:** Inclusion criteria.

Participants	Adult cancer patients
Interventions	Tai Chi Qigong Wing Chun Baduanjin
Comparator	Control interventions Waitlist controls Usual care groups
Outcome measure	Level of fatigue
Type of studies	Randomised controlled clinical trials

### 3.2 Selection Criteria

#### 3.2.1 Inclusion Criteria

#### 3.2.2 Excluded Studies

Studies were excluded if they (i) were published in languages other than English, (ii) did not use a validated tool to measure fatigue, (iii) were not full‐text articles or (iv) did not provide primary outcome data.

### 3.3 Study Selection

Two independent reviewers screened the titles and abstracts of the extracted studies. Full texts of the potential studies were then analysed. A summary of the included studies is given in Table 2 [10, 11, 12, 13, 14, 15, 16, 17, 18, 19, 20, 21, 22, 23, 24, 25].

**TABLE 2:** Summary of the included studies.

Year	Author	Country/region	Type of cancer	*N*	Mean age	Intervention	Timing of intervention	Duration (weeks)	Length per session × frequency (no. of sessions per week)	Control	Outcome measures	Time of measurement	Results
2010	Oh	Australia	Malignancy of any stage	162 (E:79 C:83)	60	Qigong	Not limited	10	1.5 h × 2	Usual care	FACT‐F	Baseline, post‐intervention	The experimental group showed significantly greater improvements in scores on fatigue (*p* < 0.001), as measured using the FACT‐F, than the control group at 10 weeks
2013	Chen	China	Breast	96 (E:49 C:47)	45	Qigong	Undergoing radiotherapy	5–6	40 min × 5	Waitlist control	BFI	Baseline, in the middle of RT, in the last week of RT, 1 month and 3 months after the end of RT	Among women who had elevated depressive symptoms at the start of radiotherapy, those in the experimental group reported less fatigue (*p* < 0.01) than those in the control group at 3 months after the end of RT. No significant between‐group differences were observed for those with low baseline depressive symptoms at any assessment point
2014	Campo	USA	Prostate	40 (E:20 C:20)	E:72C:73	Qigong	Completed treatment	12	1 h × 2	Stretch control	FACIT‐F	Baseline, 1 week post‐intervention	The experimental group showed significantly greater improvements in the FACIT‐F scores (*p* = 0.02) than the control group
2015	Larkey	USA	Breast	101 (E:49 C:52)	59	Tai Chi Easy + Qigong	Completed treatment	12	1 h × 2 in first 2 weeks, then once a week +30 min × 5 (home practice)	Sham Qigong	FSI	Baseline, post‐intervention, 3 months post‐intervention	Fatigue decreased significantly in the experimental group compared with the control group at post‐intervention (*p* = 0.005) and at the 3‐month follow‐up (*p* = 0.024)
2015	Thongteratham	Thailand	Breast	30 (E:15 C:15)	NR	Tai Chi + Qigong	Completed treatment	12	1 h × 3	Usual care	FSI	Baseline, 6 weeks, 12 weeks	The experimental group showed significant improvements in fatigue compared with the control group between the 6th week and 12th week after controlling for baseline data (*p* = 0.002)
2016	Zhang	China	Lung	96 (E:48 C:48)	63	Tai Chi	Undergoing chemotherapy	12	1 h × 3–4 (every other day)	Low‐impact exercise	MFSI‐SF	Baseline, before the third course of chemotherapy, at the end of the fourth course of chemotherapy	The experimental group had a significantly lower MFSI‐SF total score than the control group (*p* < 0.05) at 6 weeks and 12 weeks (Remarks: no time effect was analysed; only between‐group differences were reported)
2017	Chuang	Taiwan	Non‐Hodgkin lymphoma	100 (E:50 C:50)	E:56 C:65	Qigong	Undergoing chemotherapy	3	50–75 min × 7	Usual care	BFI	Baseline, 1 week, 2 weeks, 3 weeks	The experimental group exhibited significant decreases in fatigue intensity (*p* < 0.01) and fatigue interference (*p* < 0.01) compared with the control group
2017	Irwin	USA	Breast	90 (E:45 C:45)	60	Tai Chi Chih	Completed treatment	12	2 h × 1	Cognitive behavioural therapy	MFSI	Baseline, 2 months before baseline, post‐intervention, 6 months and 15 months post‐intervention	There were no between‐group differences in the change in fatigue from baseline to 3, 6 and 15 months post‐intervention
2017	McQuade	USA	Prostate	76 (E:26 C1:26 C2:24)	E: C:	Tai Chi + Qigong	Undergoing radiotherapy	20	40 min × 3	Light exercise (C1) and waitlist control (C2)	BFI	Baseline, in the middle of RT, in the last week of RT, 1 month and 3 months after the end of RT	There were no between‐group differences in the domain of fatigue at any time point
2018	Zhou	China	NPC	114 (E:57 C:57)	NR	Tai Chi	Undergoing chemotherapy and radiotherapy	13	1 h × 5	Usual care	MFSI‐SF	Baseline, post‐intervention	After chemoradiotherapy, the experimental group exhibited lower scores in the MFSI‐SF total scale and three negative subscales (general, physical and emotional fatigue) and a higher score in the vigour subscale than the control group (*p* < 0.01 for all)
2019	Lu	China	Colorectal	90 (E:45 C:45)	E:56 C:55	Qigong	Completed surgical resection with no history of chemotherapy	24	80 min × 5–7	Usual care	BFI	Baseline, 12 weeks, 24 weeks	Significant differences in cancer‐related fatigue scores were observed for the time and time‐group interaction (*p* < 0.01), but not for the inter‐group comparison
2020	Cheng	Taiwan	Malignancy of any stage	52 (E:27 C:25)	NR	Qigong	Completed treatment	12	2 h × 1	Mindfulness	CFS	Baseline, post intervention, 3‐month follow‐up	A significant reduction was found in cancer‐related fatigue scores from baseline to post‐intervention (*p* = 0.026) and to the 3‐month follow‐up (*p* = 0.017) in the Qigong group. A similar trend was also observed in the mindfulness group
2021	Molassiotis	Vietnam	Lung	156 (E:78 C:78)	E:58 C:56	Qigong + usual care	At least 4 weeks since treatment completion	12	90 min × 2 in the first 2 weeks; at least 30 min × 5 in the following 4 weeks; followed by 6 weeks of self‐practice	Usual care	FACT‐F	Baseline, 6 weeks, 12 weeks	No significant interaction effect was found between group and time for the overall symptom cluster and for fatigue and anxiety. However, a significant trend of within‐group changes in improvement in fatigue was observed (*p* = 0.004) in the Qigong group from baseline assessment to the end of intervention in the 6th week
2022	Wei	China	Breast	70 (E:35 C:35)	E:52 C:55	Baduanjin	Undergoing chemotherapy	12	30 min × 5	Face‐to‐face interview and education on self‐care	MFSI‐SF‐C	Baseline and 4 weeks, 8 weeks and 12 weeks post‐intervention	Exercise had a negative effect on fatigue (*p* < 0.001) but a significant positive effect on cognition (*p* < 0.001)
2022	Wen	China	Nasopharyngeal	88 (E:44 C:44)	E:46 C:47	Baduanjin	Completed radiotherapy and chemotherapy	12	40 min × 5	Usual care	MFI‐20	Baseline, 12 weeks	In the intention‐to‐treat analysis, the decreases in global MFI‐20, psychological and physical fatigue scores were significantly greater in the Baduanjin group than in the control group, but the difference in mental fatigue scores between the two groups was not significant. The time and effect interaction analysis did not show significant results
2022	Yao	Australia & China	Breast	72 (E:36 C:36)	E:45 C:49	Tai Chi	Completed treatment	8	1 h × 2	Usual care	BFI	Baseline, post‐intervention, 4‐week follow‐up	Tai Chi group participants showed a significant reduction in fatigue (*p* < 0.001) at immediately post‐intervention and in the follow‐up

Abbreviations: BFI, brief fatigue inventory; CFS, Cancer Fatigue Scale; CL, control; E, experimental; FACIT‐F, The Functional Assessment of Chronic Illness Therapy—Fatigue; FACT‐F, The Functional Assessment of Cancer Therapy—Fatigue; FSI, fatigue symptom inventory; MFI‐20, multidimensional fatigue inventory; MFSI, multidimensional fatigue symptom inventory; MFSI‐SF, multidimensional fatigue symptom inventory short form; MFSI‐SF‐C, multidimensional fatigue symptom inventory short form (chinese version); NR, not reported; P, placebo; RT, radiotherapy.

### 3.4 Data Extraction

Data on the key characteristics of the included studies were extracted by two independent reviewers and recorded in Excel format. The key characteristics were the countries of the studies, type of cancer, number of participants and their mean age, type of intervention, control group intervention, timing of the interventions, duration and frequency of the interventions, tools used for outcome measurement, timing of the measurements and results of the study. If the raw data were not published in the article, the authors were contacted where possible. Disagreements were resolved with team discussion.

### 3.5 Methodological Quality

The risk of bias of the studies was assessed using the most updated version 2 of the Cochrane risk‐of‐bias tool (RoB 2) for randomised trials, which was published in 2019 [26]. RoB 2 analyses the risk of bias across five domains: randomisation process, deviations from intended interventions, missing outcome data, measurement of the outcome, selection of the reported result, and overall risk of bias. Two assessors analysed the studies independently, and disagreements were settled via discussion.

### 3.6 Statistical Analyses

ReviewManager (RevMan version 5.4.1) by Cochrane was used for statistical analysis. Random‐effects models were used for the main meta‐analysis to synthesize the outcomes reported in the included RCTs. This statistical model is used as it accounts for variability across studies and is preferred when heterogeneity is moderate or high. For each outcome, the pre‐test and post‐test sample sizes and means and standard deviations (SDs) of the outcomes in each group were extracted. When these data were not available, data in the form of confidence intervals and standard errors were converted into mean (±SD) changes in scores using OpenMeta[Analyst] software developed by Brown University in the United States. As different outcome measures were used in the selected studies and higher scores in some outcome measure tools indicate more severe fatigue, the mean differences in outcome scores that were inversely related to fatigue were multiplied by −1.

If a study involved more than one intervention group, only the intervention involving martial arts training was included in the analysis [18]. The SMD, defined as the absolute mean difference divided by SD, with 95% CI and summary effect estimate was then generated. Cohen's d values of 0.2, 0.5, and 0.8 were defined as small, medium, and large pooled effect sizes, respectively, based on Cohen's rule of thumb.

Two pooled effect sizes are presented in the Results section: (1) immediate post‐intervention effect and (2) long‐term effects (≥ 12 weeks after the end of intervention).

### 3.7 Assessment of Heterogeneity

High heterogeneity could affect the internal validity and interpretation of pooled results. Thus, *I* ^2^ statistics, a quantitative measure, was analysed. *I* ^2^ values of 25%, 50%, and 75% were considered to represent low, moderate, and high heterogeneity, respectively.

To reduce the impact of heterogeneity among studies, sensitivity analysis was also performed to explore the influence of individual studies on the overall estimate, as shown in Table 3. In the analysis, the studies of interest were eliminated one at a time, and the respective SMDs with 95% CIs were calculated. ReviewManager (RevMan version 5.4.1) was used to perform this analysis.

**TABLE 3:** Sensitivity analysis.

Excluded study	SMD [CI]	*Z*	*p*	*I* ^2^
2010 Oh	−0.19 [−0.55, 0.16]	1.06	0.29	89%
2013 Chen	−0.25 [−0.62, 0.11]	1.35	0.18	90%
2014 Campo	−0.25 [−0.61, 0.10]	1.40	0.16	90%
2015 Larkey	−0.21 [−0.57, 0.15]	1.13	0.26	90%
2015 Thongteratham	−0.22 [−0.58, 0.13]	1.22	0.22	90%
2016 Zhang	−0.27 [−0.63, 0.09]	1.46	0.14	90%
2017 Chuang	−0.07 [−0.30, 0.16]	0.58	0.56	75%
2017 Irwin	−0.29 [−0.64, 0.05]	1.67	0.09	89%
2017 McQuade	−0.24 [−0.60, 0.12]	1.32	0.19	90%
2018 Zhou	−0.28 [−0.63, 0.07]	1.56	0.12	89%
2019 Lu	−0.27 [−0.63, 0.09]	1.48	0.14	90%
2020 Cheng	−0.25 [−0.61, 0.11]	1.34	0.18	90%
2021 Molassiotis	−0.23 [−0.61, 0.14]	1.23	0.22	90%
2022 Wei	−0.21 [−0.57, 0.15]	1.14	0.25	90%
2022 Wen	−0.23 [−0.59, 0.14]	1.22	0.22	90%
2022 Yao	−0.22 [−0.59, 0.14]	1.20	0.23	90%
None	−0.23 [−0.57, 0.11]	1.32	0.19	89%

### 3.8 Subgroup Analysis

The following subgroup analyses were performed to evaluate the effects of martial arts training on fatigue level in cancer patients:

- Undergoing and completed cancer treatments
- Long (≥ 12 weeks) and short (< 12 weeks) intervention periods
- Lower (< 180 min per week) and higher (≥ 180 min per week) intensity interventions
- Comparisons with active and inactive controls

### 3.9 Publication Bias

For our meta‐analysis, all studies related to the study questions were collected, irrespective of the nature of the results of the studies, to generate an overall effect estimate as objectively as possible [27]. Thus, funnel plot analysis was performed.

## 4 Results

### 4.1 Selected Studies

Our search yielded 6881 relevant studies. After removing duplicates and assessing eligibility, 16 studies that fulfilled the selection criteria were included in our analysis. Details are shown in Figure 1 and Table 2.

### 4.2 Methodology Quality—Risk of Bias Assessment

**FIGURE 2:**
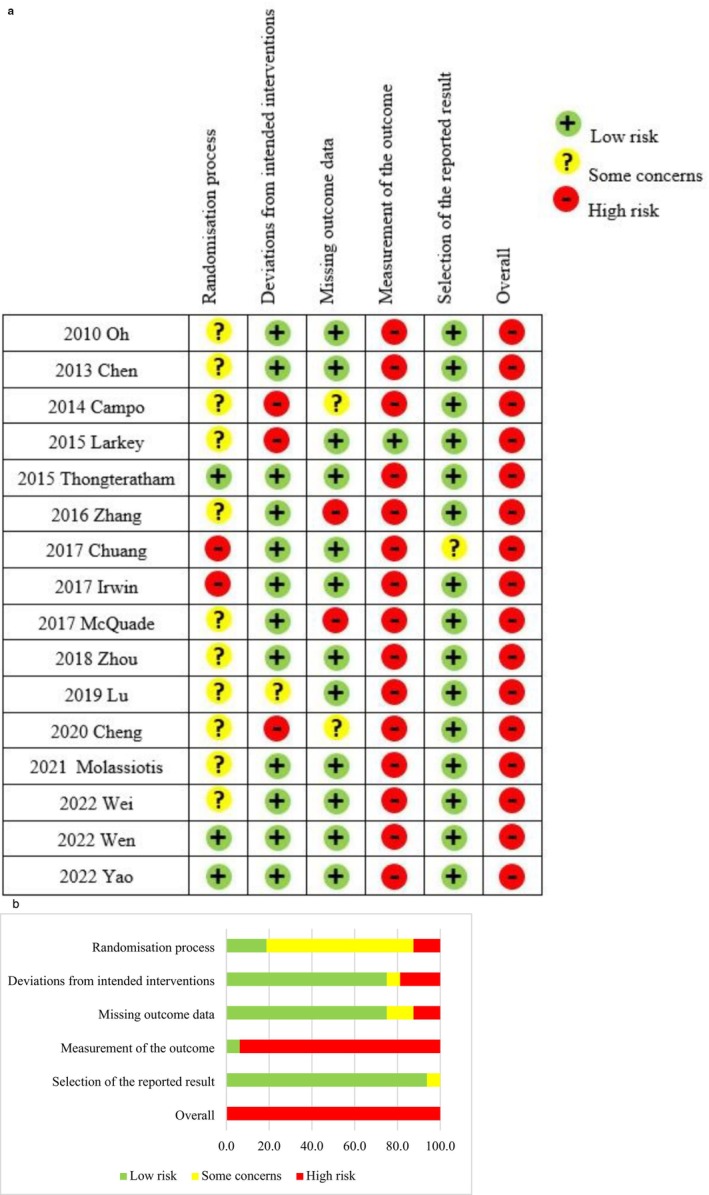
(a) Risk of bias summary: authors' judgement about each risk of bias item for each included study. (b) Risk of bias graph: authors' judgement about each risk of bias presented as a percentage across all of the included studies.

The risk of bias assessment outcome of each study is shown in Figure 2. Using the RoB2 tool, the majority of the studies were found to have a ‘high risk of bias’ in the area of ‘measurement of the outcome’, probably because in most of the studies, the outcome assessors were also the subjects as they had to self‐report their fatigue level using questionnaires. In studies that had no placebo control, all outcome assessors were aware of the intervention received. The knowledge of the intervention may have affected the results of the assessments. The updated RoB2 tool has a strict criterion in that when the assessment reveals a ‘high risk of bias’ even in one area, the overall risk of bias is rated as ‘high’. This explains why it is difficult for the included studies to not be rated as having a ‘high overall risk of bias’.

### 4.3 Study Characteristics

All of the 16 included studies were RCTs [10, 11, 12, 13, 14, 15, 16, 17, 18, 19, 20, 21, 22, 23, 24, 25]. More than half of the studies were conducted in Asia, of which nine were conducted in mainland China or Taiwan. The types of cancer that the patients had were diverse, with breast cancer being the most predominant type, present in approximately 37.5% of the included studies. Other types of cancer in the studies were prostate cancer (two studies), lung cancer (two studies), nasopharyngeal cancer (two studies), colorectal cancer (one study), and non‐Hodgkin's lymphoma (one study). Two studies included cancer patients with various malignancies. A total of 1365 patients with an age range of 45–73 years were analysed.

The intervention groups received Qigong, Tai Chi, Tai Chi Easy, or Baduanjin. Among the control groups, half of them received usual care, two were waitlist controls, three received low‐impact exercise or stretching exercise, one group received cognitive behavioural therapy, one received a mindfulness intervention, and one received patient education. In addition, Larkey et al. [13] investigated the placebo effects of martial arts training.

The timing of intervention, frequency, and duration of training and timing of measurement of treatment outcomes varied largely across the studies. The majority of the studies (nine studies) implemented martial arts training after the completion of cancer treatments. Six studies introduced martial arts training for patients while they were undergoing cancer treatments. In one study [10], no restrictions were placed on the timing of the intervention. The frequency of intervention ranged from once per week to daily. The duration of each session ranged from 30 min to 2 h. The whole duration of training also varied widely, from 3 to 24 weeks.

The outcome measures used to measure cancer‐related fatigue were the Brief Fatigue Inventory (BFI), Cancer Fatigue Scale (CFS), Functional Assessment of Chronic Illness Therapy—Fatigue (FACIT‐F), Functional Assessment of Cancer Therapy—Fatigue (FACT‐F), Fatigue Symptom Inventory (FSI), Multidimensional Fatigue Inventory (MFI‐20), Multidimensional Fatigue Symptom Inventory (MFSI), MFSI—Short Form (MFSI‐SF), and MFSI‐SF—Chinese Version (MFSI‐SF‐C).

### 4.4 Immediate Post‐Intervention Effect

Of the 16 included studies, eight reported favourable effects of martial arts training in terms of alleviating fatigue in cancer patients compared with the control interventions, while seven studies showed no between‐group differences. Chen et al. [11] showed that cancer patients with more depressive symptoms at baseline exhibited a more significant reduction in fatigue symptoms.

**FIGURE 3:**
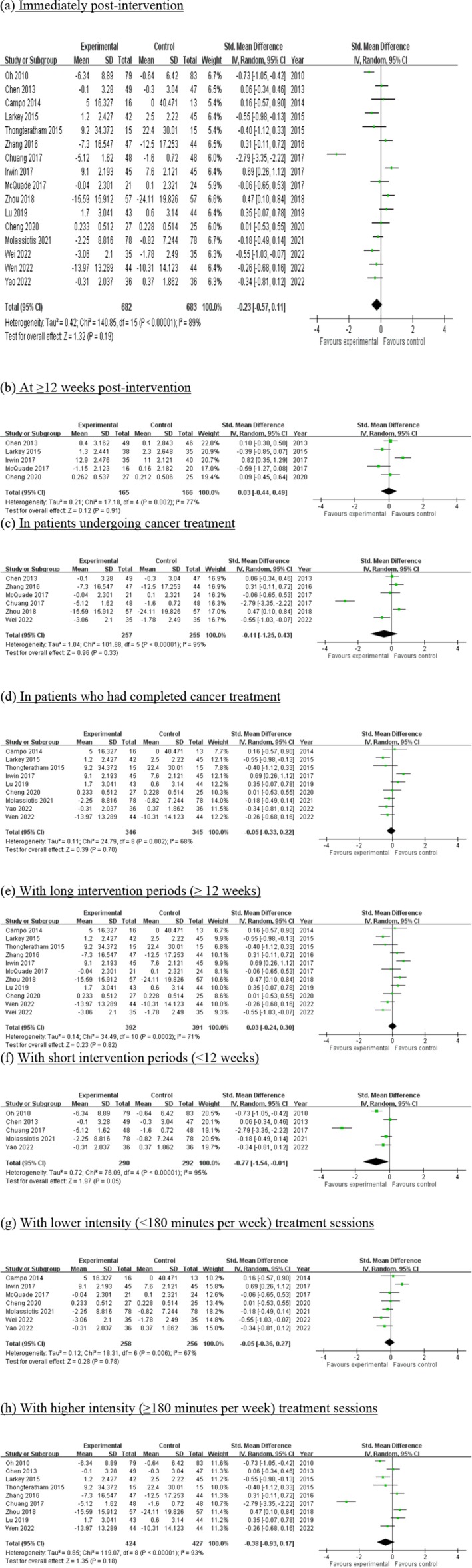
Forest plot of the effects of martial arts training on fatigue: (a) Immediately post‐intervention. (b) At ≥ 12 weeks post‐intervention. (c) In patients undergoing cancer treatment. (d) In patients who have completed cancer treatment. (e) With long intervention periods (≥ 12 weeks). (f) With short intervention periods (< 12 weeks). (g) With lower intensity (< 180 min per week) treatment sessions. (h) With higher intensity (≥ 180 min per week) treatment sessions. (i) Compared with active controls. (j) Compared with inactive controls.

Overall, the pooled effect size was small and not statistically significant (SMD: −0.23, 95% CI: −0.57 to 0.11, *p* = 0.19). The *I* ^2^ statistics indicated high heterogeneity (*I* ^2^ = 89%, df = 15, *p* < 0.01) among the studies. The forest plot is shown in Figure 3a.

### 4.5 Long‐Term Effects (≥ 12 Weeks After the End of Intervention)

Nearly one third of the included studies investigated the long‐term effects of the intervention. The pooled effect was almost neutral and statistically non‐significant (SMD: 0.03, 95% CI: −0.44 to 0.49, *p* = 0.91). The *I* ^2^ statistics indicated high heterogeneity among the studies (*I* ^2^ = 77%, df = 4, *p* = 0.002). The results are shown in Figure 3b.

### 4.6 Subgroup Analysis

#### 4.6.1 Patients Who Had Been Undergoing Cancer Treatment vs. Patients Who Had Completed Cancer Treatment

In the study by Oh et al. [10], both patients who were undergoing cancer treatment and those who had completed cancer treatment were included. Thus, this study was not included in our subgroup analysis. In the six studies in which patients were undergoing cancer treatments, as shown in Figure 3c, martial arts training showed more favourable outcomes with a small‐to‐medium pooled effect size that was statistically non‐significant (SMD: −0.41, 95% CI: −1.25 to 0.43, *p* = 0.33). The *I* ^2^ statistics indicated high heterogeneity among the studies (*I* ^2^ = 95%, df = 5, *p* < 0.001).

In the remaining nine studies in which patients had completed cancer treatment, the outcomes were neutral and statistically non‐significant (SMD: −0.05, 95% CI: −0.33 to 0.22, *p* = 0.7). The *I* ^2^ statistics indicated moderate‐to‐high heterogeneity among the studies (*I* ^2^ = 68%, df = 8, *p* = 0.002), as shown in Figure 3d.

#### 4.6.2 Long (≥ 12‐Week) and Short (< 12‐Week) Intervention Periods

The outcomes of studies with long intervention periods of ≥ 12 weeks and short intervention periods of < 12 weeks were analysed. As shown in Figure 3e, in 11 studies that had long intervention periods, the pooled statistics did not favour martial arts training and showed a statistically non‐significant effect size (SMD: 0.03, 95% CI: −0.24 to 0.30, *p* = 0.82). The *I* ^2^ statistics indicated moderate heterogeneity among the studies (*I* ^2^ = 71%, df = 10, *p* = 0.0002).

In contrast, five studies with short intervention periods showed favourable outcomes, with a medium‐to‐large pooled effect size that was statistically significant (SMD: −0.77, 95% CI: −1.54 to −0.01, *p* = 0.05). The *I* ^2^ statistics indicated high heterogeneity among the studies (*I* ^2^ = 95%, df = 4, *p* < 0.01). The forest plot is shown in Figure 3f.

#### 4.6.3 Lower (< 180 min per Week) and Higher (at Least 180 min per Week) Intensity Interventions

Frequency is an important parameter when prescribing exercise interventions. In this meta‐analysis, we defined a training duration of less than 180 min per week as lower intensity and of at least 180 min per week as higher intensity based on our clinical practice experience. As shown in Figure 3g, seven studies that implemented lower intensity interventions showed a neutral pooled effect size that was not statistically significant (SMD: −0.05, 95% CI: −0.36 to 0.27, *p* = 0.78). The *I* ^2^ statistics indicated moderate heterogeneity among the studies (*I* ^2^ = 67%, df = 6, *p* = 0.006).

For the nine studies that implemented higher intensity interventions, the pooled effect size was small and not statistically significant (SMD: −0.38, 95% CI: −0.93 to 0.17, *p* = 0.18), as shown in Figure 3h. The *I* ^2^ statistics indicated high heterogeneity among the studies (*I* ^2^ = 93%, df = 8, *p* < 0.001).

#### 4.6.4 Comparisons With Active and Inactive Controls

Of the included studies, some compared the effects of martial arts training with those of active interventions, e.g., line‐dancing, sham Qigong, low‐impact exercise and cognitive behavioural therapy [13, 15, 17, 18], while others compared them with the effects of inactive controls, including waitlist control, usual care and health education [10, 11, 14, 16, 18, 19, 20, 22, 23, 24, 25]. As shown in Figure 3i, six studies compared the effects of martial arts training with active controls and showed a neutral pooled effect size that was not statistically significant (SMD: 0.11, 95% CI: −0.27 to 0.50, *p* = 0.78). The *I* ^2^ statistics indicated moderate heterogeneity among the studies (*I* ^2^ = 71%, df = 5, *p* = 0.004).

In the 11 studies that compared the effects with inactive controls, the pooled effect size was small and not statistically significant (SMD: −0.39, 95% CI: −0.82 to 0.05, *p* = 0.08), as shown in Figure 3j. The *I* ^2^ statistics indicated high heterogeneity among the studies (*I* ^2^ = 91%, df = 10, *p* < 0.001). In sum, no statistically significant results were found in the subgroup analysis for comparisons with inactive or active controls.

### 4.7 Publication Bias

**FIGURE 4:**
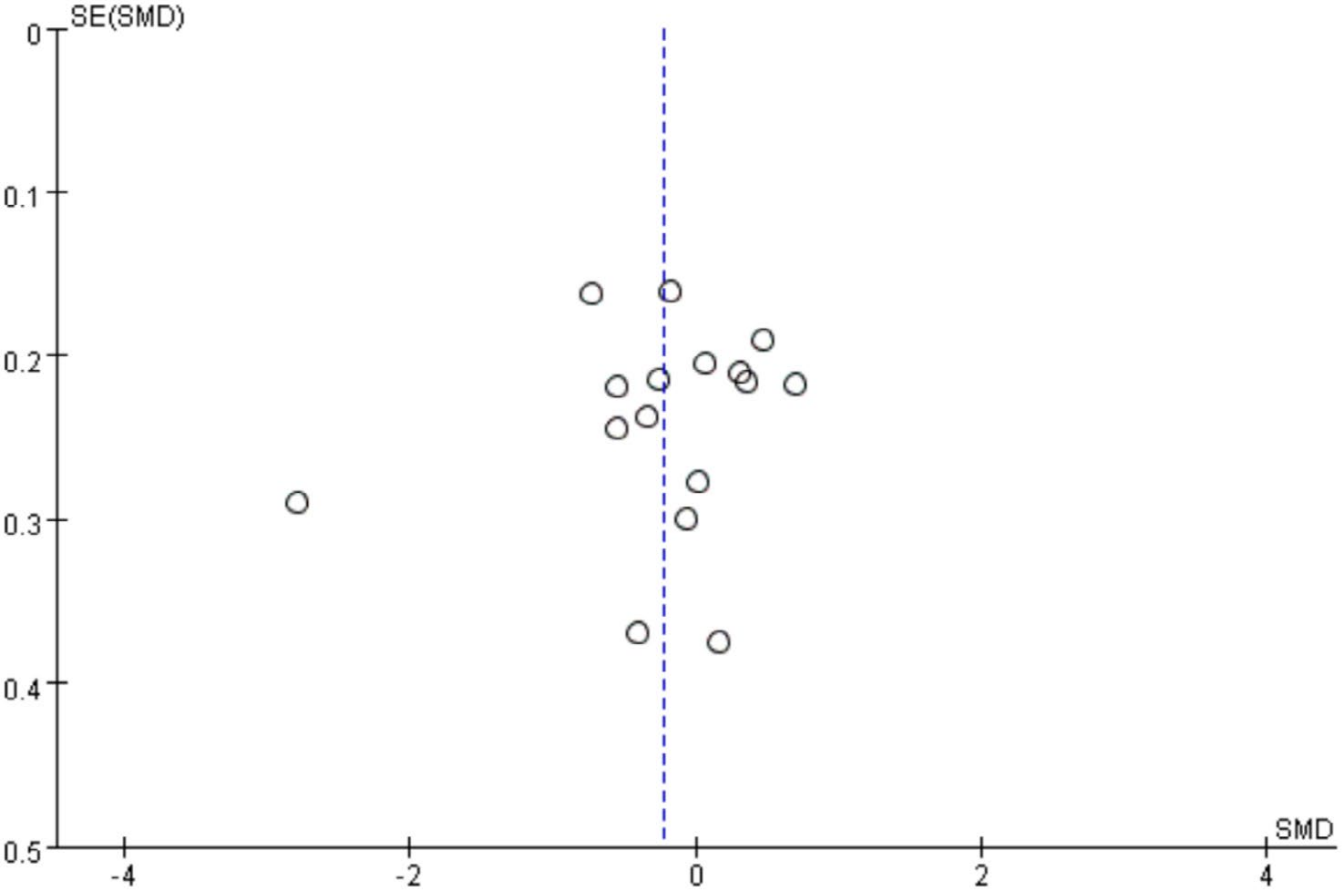
Funnel plot.

A funnel plot analysis was performed, and the results are shown in Figure 4. Small‐scale studies with nonsignificant effects may be more likely to be unpublished or missed than other studies [27]. The funnel plot in Figure 4 displays asymmetry, suggesting potential publication bias. Our sensitivity analysis results revealed that no individual study significantly affected the overall estimate. This indicates that the results are statistically robust (Table 3).

## 5 Discussion

The effects of martial arts training on general cancer patients have been evaluated in a few meta‐analyses [7, 8, 9]. Nonetheless, to the best of our knowledge, this is the first meta‐analysis to analyse the effects from the perspective of developing a clinical exercise guideline. Instead of analysing the effects on fatigue using various outcome measures, we analysed the effects from a clinical perspective, i.e., the effects of training periods and intensity and timing of the intervention, and the long‐term effects.

In terms of the methodological quality of the included studies, the overall risk of bias of the included studies was high, as measured using the RoB2. The majority of the studies were rated as having a ‘high risk of bias’ in the domain of ‘measurement of outcome’ except for the study by Larkey et al. [13] that included a sham group. In studies without a placebo control, the subjects were aware of the treatment they received. Thus, knowledge of the intervention might have affected the results of assessments conducted using self‐rated questionnaires. Accordingly, the overall risk of bias of studies was high.

Overall, statistical analysis showed a high level of heterogeneity among studies, which can be explained by several reasons. First, the types of cancer and baseline characteristics of the disease groups varied across the studies. Second, a wide variety of outcome measurement tools were used in the included studies. The scores of fatigue scales are converted to standardised mean differences, for comparison across different fatigue outcome measuring tools, through statistical methods. However, the internal validity was inevitably lowered. Third, the comparison groups also varied among the studies. Lastly, the inclusion and exclusion criteria differed between the studies. The substantial levels of heterogeneity thus observed in this meta‐analysis are consistent with those reported in the meta‐analyses conducted by Wayne et al. [7] and Yin et al. [9].

The considerable level of heterogeneity of this meta‐analysis may make the results more generalizable among different cancer populations, irrespective of their stages and cancer treatments. However, the internal validity of the results may be lowered. This meta‐analysis may serve as a foundation for developing future interventions. In future studies, prospective randomised trials studying the effects of Chinese martial arts training, with different intensities and durations, on homogenous patient groups of cancer types, stages of cancer, and cancer treatments should be conducted. Furthermore, the future prospective trials should incorporate some widely adopted outcome measures for fatigue, for instance, FACT‐F and EORTC‐QLQ‐C30.

To the best of our knowledge, previous meta‐analyses did not analyse the effect of martial arts training on cancer‐related fatigue from the perspective of training duration. Interestingly, interventions with shorter intervention periods, which were defined as less than 12 weeks in this meta‐analysis, reported more favourable outcomes than studies with longer intervention periods. One reason for this result may be that adherence to exercise training may have decreased as the intervention period increased. Interventions with shorter intervention periods are often more intense and structured, putting focus on specific short‐term goals. The participants' engagement in programmes is usually higher at the beginning of interventions, and attrition rates may be lower in shorter interventions. These may bring more significant immediate treatment effects. One of the main difficulties in carrying out exercise programmes is ensuring compliance with the interventions. Better adherence to exercise programmes may yield better treatment outcomes. Therefore, when designing exercise programmes for cancer patients, clinicians should find ways to effectively monitor and improve patients' exercise compliance.

Ormel et al. [28] conducted a systematic review of the predictors of adherence to exercise interventions during and after cancer treatments. The results revealed that proximity of rehabilitation facility, extensive exercise history, and fewer exercise limitations are positive predictors of exercise adherence. In our selected studies, 11 studies involved home exercise training, but only four of these recorded compliance with the training [12, 22, 23, 25]. In addition, of the 16 included studies, only seven reported class attendance rates or completion rates [11, 12, 13, 16, 17, 18, 22]. In future studies, researchers should also analyse outcomes related to exercise compliance and adherence rates as compliance may be an important predictor of the treatment outcome.

Another reason for the more significant effects in those interventions with shorter periods is that the influence of external factors is less. As cancer conditions may progress, patients' health conditions and cancer treatments may change with time. With a longer intervention period, the change in the condition of the disease may be more pronounced, making the treatment effects less detectable.

We further conducted subgroup analysis to evaluate the effects of martial arts training from the perspective of different intensities of the intervention. In exercise interventions, the frequency, intensity, time, and type of exercise are vital factors that influence outcomes and need to be monitored and modulated by clinicians. Surprisingly, the interventions implemented at a higher intensity (at least 180 min per week) were not superior to those implemented at a lower intensity in terms of their effects on cancer‐related fatigue, as the difference was not statistically significant. More frequent treatment sessions require more dedication from the participants. As exercise completion and adherence rates were not reported in most of the studies, the effects of different exercise frequencies on the reduction in cancer‐related fatigue remain inconclusive.

Previous studies have proposed several possible mechanisms by which martial arts training, e.g., Tai Chi, help to alleviate cancer‐related fatigue. These include enhancement of vagal modulation, improved regulation of the hypothalamic–pituitary–adrenal axis, reduction in cytokine levels, improvement in muscle strength [15], reduction in the cortisol level [29], reduction in cellular inflammatory responses and reduction in the expression of genes that encode proinflammatory mediators [30]. To further confirm the relationship between cancer‐related fatigue and the effects of martial arts training, researchers are recommended to include biomarker analysis in future research. As reported previously, the overall risk of bias of the included studies was high because of the use of self‐reported questionnaires to rate subjects' own perceived fatigue level. Incorporating biomarker analysis could augment the research findings and provide a quantitative and objective perspective on measuring cancer‐related fatigue.

Previous meta‐analyses did not analyse the effect of martial arts training on cancer‐related fatigue from the perspective of the timing of the intervention. Our subgroup analysis showed that martial arts training was more effective in patients who were undergoing cancer treatment than in those who had completed cancer treatment, although the difference was not statistically significant. This difference may be because patients who are undergoing treatment may experience a higher level of cancer‐related fatigue than those who have finished their treatment [15]. This finding is consistent with the results of the study by Hofman et al. [31] who reported that approximately 90% of cancer patients had cancer‐related fatigue during cancer treatment, whereas the percentage decreased to 30% among patients who had completed their treatment. Yin et al. [9] reported that the reduction in cancer‐related fatigue was greater in patients who had higher fatigue levels at baseline. The change in fatigue level may thus be more prominent when martial arts interventions are implemented during cancer treatment than when they are implemented after the treatment is completed.

In contrast, the meta‐analysis conducted by Speck et al. [32] revealed that studies that administered the exercise intervention during cancer treatment did not show a favourable effect on cancer‐related fatigue, whereas a clinically moderate benefit was found in studies that implemented the intervention after the completion of cancer treatment. Despite the contradicting results reported in the literature, as some cancer treatments require the patients to stay in the hospital for nearly a day, martial arts training may be administered as an adjunct treatment, as in the study by Zhang et al. [15]. Apart from the physical benefits to patients, the implementation of group exercise training into daily care for oncology cases may also enhance psychological support and bonding among the patients and improve the treatment outcomes. Mustian et al. [33] performed a meta‐analysis to compare the effects of pharmaceutical, psychological, and exercise interventions on cancer‐related fatigue. The results showed that exercise and psychological interventions are effective in reducing cancer‐related fatigue during and after cancer treatments and are better than pharmaceutical options. Group martial arts training, with both an exercise component and psychological support, implemented during cancer therapy may thus be an ideal intervention for clinicians to administer.

Of the studies included in this meta‐analysis, only Larkey et al. [13] compared the outcome with placebo controls. As mentioned previously, subjects' awareness of the intervention they were receiving may have affected their perception of the outcomes. Another limitation is the qualitative nature of the outcome measures, as fatigue is a subjective experience. Implementation of a placebo control would have reduced the risk of bias, especially in the area of ‘measurement of the outcome’. Therefore, it is recommended that a placebo control group be included in future studies involving subjective measurement of cancer‐related fatigue. However, the successful implementation of a sham group may not be feasible, especially when subjects have previous knowledge of Chinese martial arts.

In our subgroup analysis, we pooled the effects of martial arts training compared with active and inactive controls. Both control groups showed non‐significant differences relative to the training group. In the studies that adopted active control groups, the exercise interventions mostly included low‐impact exercise. In 2019, Campbell et al. [34] published an exercise guideline for cancer patients. They recommended that participation in moderate‐intensity aerobic training three times per week could significantly reduce cancer‐related fatigue both during and after treatment. Moreover, aerobic combined with moderate‐intensity resistance training performed 2–3 times per week or twice weekly moderate‐intensity resistance training may also be effective in reducing cancer‐related fatigue, especially in prostate cancer patients. Future studies should compare the effects of martial arts training with moderate‐intensity exercise to determine whether martial arts training is superior to other forms of exercise training for cancer patients.

Martial arts training is an Eastern form of exercise. The concept of flow of energy, namely ‘Qi’, may not be easily understood by Westerners. Tai Chi, Baduanjin, and Qigong are all forms of martial arts training with some similarities and differences. Tai Chi consists of elements of meditation, body awareness, and breathing [19], with low‐to‐moderate‐intensity physical activity, and can be considered as an additional form of aerobic exercise that can boost aerobic capacity [35]. There are different forms of Tai Chi styles, for example, Chen and Yang. Qigong is a mindfulness exercise that typically involves slow, flowing movements coordinated with deep rhythmic breathing, meditation, concentration, and relaxation. It cultivates mind–body interaction and enhances ‘Qi’, the life essence and energy within our body [36, 37]. Goulin New Qigong and Chan‐Chuang Qigong are common Qigong exercises prescribed medically [11, 16]. Baduanjin is also considered a type of Qigong exercise. In addition to the component of breathing for the regulation of ‘Qi’, Baduanjin contains eight distinct and smooth movements and is considered a light‐to‐moderate‐intensity aerobic exercise [24]. These different forms of martial arts training may have different effects on patients with different cancer types. Cultural differences may also lead to different understandings and interpretations of the exercises.

There are challenges in conducting studies involving exercise training as accuracy in performing exercise as prescribed is often difficult to attain. For research that involves home practice or self‐practice and assumes a high level of compliance, subjects may not perform the exercise accurately. This is a common challenge faced by clinicians. With the advances in instant messaging applications, researcher may consider asking subjects to take photos or videos of their self‐practice for researchers to understand how well they are practicing at home. In the study conducted by Wen et al. [24], the WeChat app, an instant message application, was used to send video demonstrations of Baduanjin exercise, and video conferences with the coach were conducted to ensure effective delivery of the intervention. This serves as a good example of the use of new technology to enhance effective and accurate delivery of the intended intervention.

## 6 Conclusion

Cancer‐related fatigue is a common problem experienced by cancer patients. Results of this current review and meta‐analysis did not indicate any significant effect of Chinese martial arts training for alleviating cancer‐related fatigue in cancer patients. However, results of our sub‐group analyses demonstrated that martial arts training administered for shorter durations (< 12 weeks) yielded a significant reduction in cancer‐related fatigue with a medium‐to‐large pooled effect size. Though non‐significant results noted, some recent studies of high quality [23, 25, 38] showed that Tai Chi and Baduanjin were effective in reduction of cancer‐related fatigue on patients with breast cancer and lung cancer.

## Author Contributions

**Benjamin K. K. Lau:** conceptualization, data curation, formal analysis, investigation, methodology, project administration, software, writing – original draft. **Tai Wa Liu:** supervision, validation, writing – review and editing. **Shamay S. M. Ng:** supervision, validation, writing – review and editing. **William W. N. Tsang:** supervision, validation, writing – review and editing.

## Funding

The authors have nothing to report.

## Ethics Statement

The authors have nothing to report.

## Consent

The authors have nothing to report.

## Conflicts of Interest

The authors declare no conflicts of interest.

## Data Availability

The data that support the findings of this study are available from the corresponding author upon reasonable request.
